# A Novel BAT3 Sequence Generated by Alternative RNA Splicing of Exon 11B Displays Cell Type-Specific Expression and Impacts on Subcellular Localization

**DOI:** 10.1371/journal.pone.0035972

**Published:** 2012-04-25

**Authors:** Nadine Kämper, Jörg Kessler, Sebastian Temme, Claudia Wegscheid, Johannes Winkler, Norbert Koch

**Affiliations:** Division of Immunobiology, Institute of Genetics, University of Bonn, Bonn, Germany; International Centre for Genetic Engineering and Biotechnology, Italy

## Abstract

**Background:**

The human lymphocyte antigen (HLA) encoded BAT3/BAG6 recently attracted interest as a regulator of protein targeting and degradation, a function that could be exerted in the cytosol and in the nucleus. The *BAT3* gene was described to consist of 25 exons. Diversity of transcripts can be generated by alternative RNA splicing, which may control subcellular distribution of BAT3.

**Methodology/Principal Findings:**

By cDNA sequencing we identified a novel alternatively spliced sequence of the *BAT3* gene located between exons 11 and 12, which was designated as exon 11B. Using PCR and colony hybridization we identified six cDNA variants, which were produced by RNA splicing of *BAT3* exons 5, 11B and 24. In four examined cell types the content of *BAT3* splice variants was examined. Most of the cDNA clones from monocyte-derived dendritic cells contain exon 11B, whereas this sequence was almost absent in the B lymphoma Raji. Exon 5 was detected in most and exon 24 in approximately half of the cDNA clones. The subcellular distribution of endogenous BAT3 largely correlates with a cell type specific splicing pattern. In cells transfected with BAT3 variants, full-length and Δ24 BAT3 displayed nearly exclusive nuclear staining, whereas variants deleted of exon 11B showed substantial cytosolic expression. We show here that BAT3 is mainly expressed in the cytosol of Raji cells, while other cell types displayed both cytosolic and nuclear staining. Export of BAT3 from the nucleus to the cytosol is inhibited by treatment with leptomycin B, indicating that the Crm1 pathway is involved. Nuclear expression of BAT3 containing exon 11B suggests that this sequence plays a role for nuclear retention of the protein.

**Conclusions/Significance:**

Cell type-specific subcellular expression of BAT3 suggests distinct functions in the cytosol and in the nucleus. Differential expression of BAT3 variants may reconcile the multiple roles described for BAT3.

## Introduction

The Human Lymphocyte Antigen (HLA) locus on chromosome 6 is subdivided into a class I, II and III region. While the class I and II regions contain genes encoding HLA peptide receptors, the densely gene packed class III region is strongly associated with inflammatory immune responses, autoimmune diseases and other non-immune functions [1], [2]. A group of genes within the class III region is located adjacent to the HLA-B locus and these genes are designated as B-associated transcripts (*BAT*) [3]. The *BAT* genes are numbered from *BAT1* to *BAT9*. The *BAT3* locus recently gained substantial interest. A first functional characterization revealed that the *Xenopus* ortholog of *BAT3* (Scythe) is a regulator of *Drosophila melanogaster* protein Reaper-induced apoptosis [4]. Binding of Scythe to Reaper is followed by release of cytochrome c from mitochondria [5]. Reaper has no vertebrate homolog. However, the Reaper-response pathway appears to be conserved in vertebrates. Inactivation of the *BAT3* ortholog in mice is associated with pronounced developmental defects in the lung, kidney, and brain, which were ascribed to dysregulation of apoptosis and cellular proliferation [6].

The BAT3 protein is rich in proline residues and shows repeated domain structures with sequence homologies to domains of other proteins. The presence of a BAG domain at the C-terminus and an ubiquitin-like domain at the N-terminus of BAT3 suggests that BAT3 (BAG6) plays a role in protein folding and proteasomal degradation [3], [7]. Interaction of *Xenopus*-derived BAT3 (Scythe) with a subunit of the proteasomal complex [8] and with the *Xenopus* elongation factor α1 (XEFIAO) indicates a role of BAT3 for degradation of cytosolic proteins [9]. Scythe is required for degradation of XEFIAO, which if accumulated in oocytes induces apoptosis. Further studies demonstrated that BAT3 binds to the acetyltransferase p300 and controls DNA damage-induced acetylation of p53 [10]. In addition, BAT3 stabilises the apoptosis-inducing factor (AIF), which relocates upon induction of apoptosis from the mitochondrial intermembrane space to the nucleus [11]. In several recent reports BAT3 was shown to regulate cytosolic protein targeting and proteasomal degradation [12], [13], [14], [15].

Adding to the diverse roles of BAT3, the protein was identified as a ligand of the cell surface NKp30 receptor, an activating member of the NK receptor family [16]. Impact on the cytolytic activity of NK cells had been demonstrated previously for another member of the BAG family, BAG4. Stimulation of NK cell activity was detected in conjunction of Hsp70 and BAG4. These molecules were found attached to exosomes and thereby released to the extracellular fluid [17].

BAG family members are detected in both, the nucleus and the cytoplasm [7]. Identification of a nuclear localization signal (NLS) in the BAT3 sequence was in agreement with detection of the polypeptide in the nucleus [18]. In order to explain intracellular shuttling of BAT3 between the nucleus and the cytosol, an N-terminal nuclear export signal was suggested [11]. At present it is not clear on what level the subcellular localization of BAT3 is regulated.

Differential pre-mRNA splicing may contribute to expanding protein diversity [19]. It is conceivable that some of the diverse roles of BAT3 could be explained by BAT3 isoforms which are generated by differentially spliced RNA. The *BAT3* mRNA of the human gene was described to be about 3.7 kb in length and transcribed from 25 exons, of which 24 are translated [3]. The intron-exon organisation of the *BAT3* gene is conserved in mammalian species [20]. Evaluation of cDNA database sequences suggests that several splice products of the human *BAT3* transcript exist, but the presence of BAT3 splice variants is rarely considered and their cellular expression has not been studied yet.

We inspected the diversity of BAT3 by identifying naturally occurring splice products. By sequencing *BAT3* cDNAs we discovered a novel alternatively spliced *BAT3* exon, located between exon 11 and 12, which we designated as exon 11B. By examining 100 *BAT3* cDNA clones, we isolated six *BAT3* variants with variable content of exon 5, 11B and 24 derived sequences. The BAT3 isoforms showed a cell type-specific expression pattern and a different subcellular distribution. The translocation of BAT3 from the nucleus to the cytosol is blocked by treatment of cells with leptomycin B, indicating Crm1-dependent nuclear export. Tissue-specific expression of BAT3 splice variants and their variable subcellular expression could be involved in the multifunctional properties reported for BAT3.

## Materials and Methods

### Ethics statement

Primary monocyte-derived DCs (moDC) were a kind gift from Dr. S. Koch, Dermatology Department, Bonn, Germany [21]. All work with human primary cells was performed in adherence to the Helsinki guidelines. The regional board of the district (cologne) approved the generation of blood samples at the local blood bank, Institute of Experimental Hematology and Transfusion Medicine, University Hospital of Bonn (approval number: 24.30.12/02/Uni Bonn-001). In accordance with the agreement of Dr. S. Koch and the Institute of Experimental Hematology and Transfusion Medicine, moDCs were generated from human monocytes isolated from buffy coat preparations obtained anonymously from the local blood bank. Buffy coats are a by-product from erythrocyte concentrates which can be used for scientific purposes without approval of the ethics committee.

### Cell lines and antibodies

The human melanoma cell line MelJuSo and the T lymphoma cell line CEMC7 were obtained from Dr. G. Moldenhauer (Deutsches Krebsforschungszentrum, Heidelberg, Germany) [22], [23]. The adenocarcinoma line HeLa (ATCC: CCL-2), the monkey kidney cell line COS-7 (ATCC: CRL-1651) and the B lymphoma cell line Raji (ATCC: CCL-86) were purchased from the ATCC Cell Biology Collection, LGC Standards (Wesel, Germany). MelJuSo, HeLa and COS-7 were grown in Dulbecco's modified Eagle medium (DMEM) containing 10% fetal calf serum and 1% antibiotics, sodium pyruvate and HEPES (PAA, Pasching, Austria). The B lymphoma cell line Raji was cultured in RPMI and dendritic cells were grown in VLE-RPMI supplemented with antibiotics and glutamine (Biochrom AG, Berlin, Germany). V5 tag-specific monoclonal antibody was purchased from Invitrogen (Karlsruhe, Germany), GADPH-specific antibody from Calbiochem (Darmstadt, Germany) and histone H3-specific antibody from Santa Cruz (Heidelberg, Germany). HLA-DR was detected with ISCR3 mAb [24]. The BAT3-specific rabbit antiserum was generated by Pineda (Berlin, Germany) using the C-terminal peptide RKVKPQPPLSDAYLSGMPAK [6] coupled to KLH as antigen. In parallel, a pre-immune serum was applied to monitor non-specific staining.

### Generation of BAT3 cDNAs

*BAT3* cDNAs were generated by RT-PCR (ThermoScript™ System, Invitrogen) with poly-A-mRNA from different cell types as templates and oligonucleotide 5′-AGGATCATCAGCAAAGGC-3′ as primer. *BAT3* was amplified using the Expand Long Template PCR System (Roche, Penzberg, Germany) with 5′-GCTAGCATGGAGCCTAATGATAGTA-3′ as forward primer and 5′-AGGATCATCAGCAAAGGC-3′ as reverse primer. The obtained PCR fragments (exons 2–25) were cloned into pcDNA3.1/V5-HisTOPO vector (Invitrogen, Karlsruhe, Germany). *BAT3* cDNA clones were tested by restriction analysis using *NheI* and *XbaI*. Preselected DNA constructs were examined by sequencing (GENterprise, Mainz, Germany).

### Characterization of *BAT3* cDNAs by PCR

**Figure 1:**
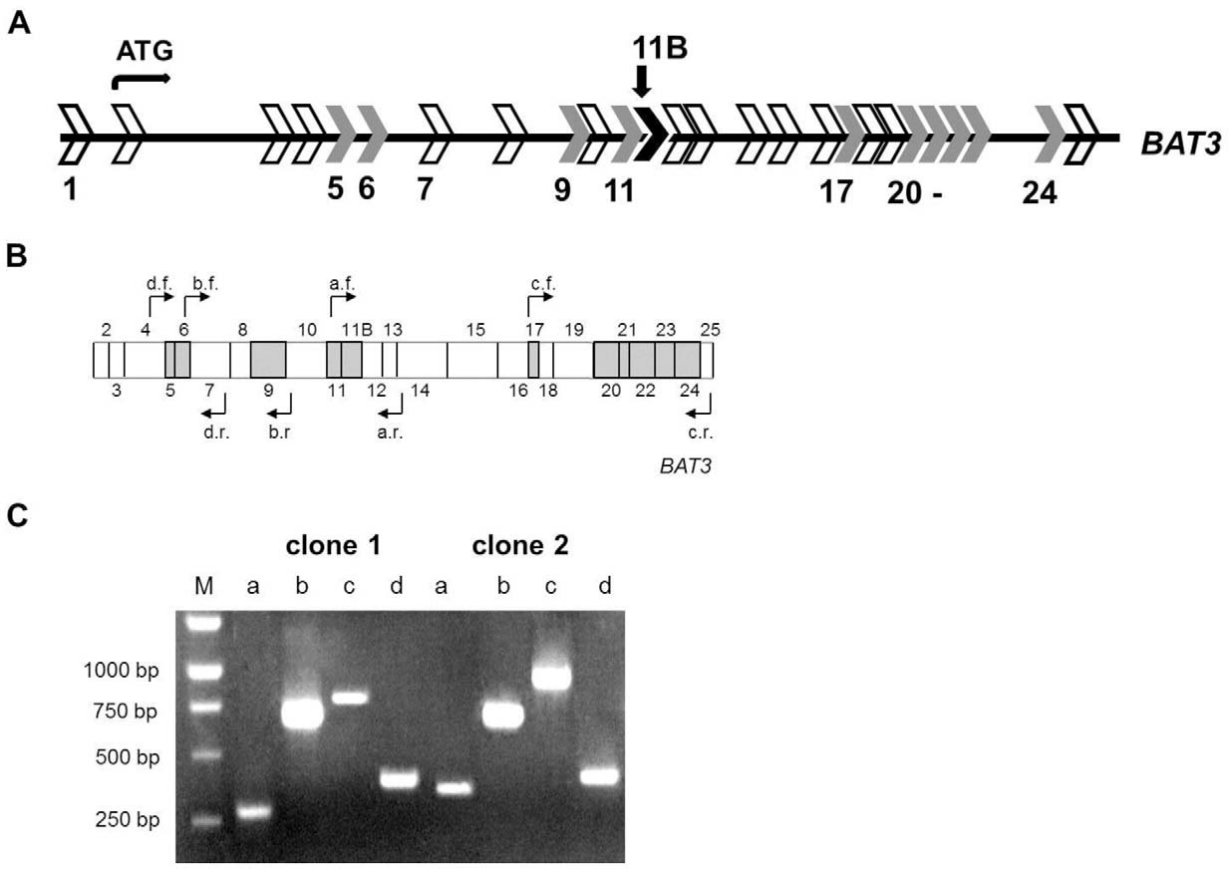
Detection of exon sequences in BAT3 cDNA clones by PCR. **A.** Exon-intron-structure of the *BAT3* gene. Exon sequences are indicated as arrows. The start codon for translation in exon 2 is indicated. Potentially spliced exons are numbered. Exon 1 and 7 exist in different fragment lengths, 247 bp or 271 bp for exon 1 (NCBI) and 236, 254 or 278 bp for exon 7 (NCBI, ENSEMBL). Exons highlighted in grey could be deleted without changing the reading frame. The novel exon 11B with a length of 108 bp is labeled by arrow. Exon 11B also can be deleted without a change of the reading frame of the adjacent exon sequences. **B.** Schematic presentation of BAT3 cDNA. Exon boundaries are indicated and protein-encoding exons are numbered from 2 to 25. Potentially spliced exons are highlighted in grey. The position of complementary primer pairs (a to d) for forward (f) and reverse (r) PCR are indicated by arrows. **C.** Primers indicated in B were used to characterize BAT3 cDNA clones. Digestion of two BAT3 cDNAs is shown for example. Lane M contains size standards with bp indicated on the left. Lanes a: PCR products of clones 1 and 2 exhibit a band of 280 bp (no exon 11B) or of 380 bp (with exon 11B). Lanes b: Both clones generate a PCR product of 660 bp, indicating the presence of exon 9. Lanes c: The sizes of PCR fragments from clone 1 and clone 2 are 800 bp or 950 bp, respectively. This size is consistent with the presence or absence of exon 24. Lanes d: The presence of exon 5 is verified in clones 1 and 2 by a PCR product of 400 bp.

To analyze the expression pattern of BAT3 splice variants in different cell types, we determined the presence or absence of alternatively spliced exon sequences based on the size of the obtained PCR fragments (Figure 1B). The presence of exons 5 and 6 was inspected with oligonucleotides 5′- GGAACCTTCAATCTTCCT -3′ (d.f.) as forward and 5′- GTTGGGTGCATTTGTTTC -3′ (d.r.) as reverse primers. Amplification of exon 9 was conducted with the oligonucleotides 5′- CATCCTTCCCCTGCG-3′ (b.f.) as forward and 5′- TTGAATGTTCATGTGCATCAT-3′ (b.r.) as reverse primers. For exons 11 and 11B we used 5′- GATTCTGGCACACAG-3′ (a.f.) as forward and 5′-CCACAAGGACTGGCTG-3′ (a.r.) as reverse primers. Exons 17 to 24 were analyzed by 5′- GGATTCTTTGGGGCC-3′ (c.f.) as forward and 5′- AGGATCATCAGCAAAGGC-3′ (c.r.) as reverse primers. PCR products were subsequently separated by agarose gel electrophoresis (Figure 1C). To identify the products containing exon 5 or the deleted sequence, a control product without exon 5 was loaded for size comparison adjacent to the examined clone onto the agarose gel. The presence of alternatively spliced exons near the N- and C-termini of BAT3 was additionally confirmed by DNA sequencing (GENterprise, Mainz, Germany).

### Colony hybridization assay

To identify BAT3 splice variants present in MelJuSo cells, we employed a colony hybridization assay to analyze 100 *BAT3* cDNA clones with exon-specific ^32^P-labelled DNA probes. To test for the presence of certain exons, transformed bacteria were inoculated on agar plates, transferred to membranes by replica plating and probed with ^32^P-labelled DNA probes. Exon-specific probes were generated using the Megaprime DNA Labelling Kit (Amersham, Freiburg, Germany). The *BAT3* templates were amplified by PCR with the following oligonucleotides: 5′-CTTCCTAGTGACGGC-3′ and 5′-CTCACTCTGAATCGG-3′ for exon 5, 5′- AGT GAGCCCCGGGTA-3′ and 5′- CTCCATCCGGGATAG-3′ for exon 6, 5′- CATCCT TCCCCTGCG-3′ and 5′- TTGAATGTTCATGTGCATCAT-3′ for exon 9, 5′- GATTCTGGCACACAG-3′ and 5′-CCAGGGTTTGGCCAT-3′ for exon 11, 5′- GCT CCACCCTCATCCAGC-3′ and 5′- CTGCGGCCGCGGAGG-3′ for exon 11B, 5′- ATC CGGATGGCAACC-3′ and 5′- AAAACTCTCCCGCACATACTC-3′ for exon 17, 5′- CGTCGTATGTCTCGTG-3′ and 5′- CTGGGGGGGATCCC-3′ for exon 20, 5′- CCC CAGCCACTTCCT-3′ and 5′- CTCCCGCTGAGGCTC-3′ for exon 21, 5′- CGGGAG AATGCTTCC-3′ and 5′- TGGGGGGACTGCAGC-3′ for exon 22, 5′- GAATGGGTC CCTATTATC-3′ and 5′- CTTGCGTCTCTTCGC-3′ for exon 23, 5′- ATGCCTGCCAAGAGA-3′ and 5′- GTATATCAGACCGGAG-3′ for exon 24. Prior to hybridization of ^32^P-labelled exon-specific DNA probes with cDNA from transformed bacteria, the replica membranes were blocked with 5 ml Ultrahyb® hybridization solution (Ambion, Austin, USA) at 42°C for 1 h. Subsequently, the ^32^P-labelled DNA probes were added to the hybridization solution and incubated over night at 42°C. After hybridization, membranes were washed with 2× SSC/ 0.1% SDS up to 0.2× SSC/ 0.1% SDS, shrink-wrapped and exposed to X-ray films at −80°C.

### Real-time PCR analysis

To quantify the expression of *BAT3* exons, real-time PCR was conducted as previously described [25]. For selective amplification of products either devoid of or containing the respective exons, the following primer pairs were used at an annealing temperature of 60°C: 5′-GACCGGAATGCCAACAGCTAT-3′ and 5′-GCCTGTTCCATGTTGATGTGAAC-3′ for exon 5, 5′-TCCTCAGACTCACCTCCCTTCT-3′ and 5′-GGCTCACTAGGAAGATTGAAGGTT-3′ for deleted exon 5, 5′-GCTCCACCCTCATCCAGCTG-3′ and 5′-CCACCATGGCCTGATGAGTG-3′ for exon 11B, 5′-AAACCCTGGGACAGCAGGTG-3′ and 5′-CGAGCCTGTGGAGGAGTGG-3′ for deleted exon 11B, 5′-TGCTTCTCTCAGAGGCTGTGAG-3′ and 5′-TGCCTGTAGCTCTCCTGAACC-3′ for exon 24, 5′-GACGCAAGCTCCGGTCTGATAT-3′ and 5′-AAGGATCATCAGCAAAGGCCC-3′ for deleted exon 24.

### Transient transfection and western blotting

COS-7 and HeLa cells were grown to 60–70% confluence and transfected using jetPEI (Biomol, Hamburg, Germany) in 6-well plates according to the instructions of the distributor. The DNA mixtures contained 2 µg of the BAT3 cDNAs. Cells were harvested 48 h after transfection and used for western blot analysis or for immunofluorescence microscopy [25].

### Immunofluorescence microscopy

For immunofluorescence microscopy cells were grown on coverslips. Cells were fixed with methanol (−20°C) for at least 5 min, washed twice with phosphate-buffered saline (PBS) and blocked using Roti-ImmunoBlock (Roth, Karlsruhe, Germany). Coverslips were incubated for 1 h at room temperature with indicated antibodies. After washing with PBS, incubation was continued for 1 h at room temperature with AlexaFluorDye-coupled secondary antibodies (Molecular Probes, Eugene, OR, USA). Coverslips were washed with PBS and mounted onto slides using PermaFluor (IMMUNOTECH, Marseille, France). Cellular staining was visualized by confocal or standard immunofluorescence microscopy (LSM-510 META or Axiophot; Zeiss, Oberkochen, Germany). Quantitative evaluation of immunofluorescence images was conducted with ImageJ (U.S. National Institutes of Health, Bethesda, MD, USA).

### Separation of nuclei and cytosol

The separation of cell nuclei and cytosol was carried out following a modified protocol of Gaynor *et al.* [26]. 2×10^7^ cells were harvested by centrifugation for 5 min at 300× g and subsequently suspended in 1 ml lysis buffer (10 mM Tris, pH 7.5, 0.25 M sucrose, 1 mM EDTA, 5 µg/ml aprotinin and 5 µg/ml leupeptin). The cell suspension was incubated for 30 min on ice and then transferred to a pre-chilled dounce homogenizer. Cells were disrupted by 25 strokes with a tight-fitting pestle. Subsequently, nuclei were pelleted for 5 min at 800× g at 4°C (p_800_). Supernatants were collected and cytosolic proteins were precipitated with ethanol_abs_. at 4°C by centrifugation at 13000× g for 30 min. Cell nuclei (p_800_) and cytosolic precipitates were resuspended in lysis buffer and subsequently subjected to SDS gel electrophoresis and western blotting.

### Leptomycin B treatment of cells

To investigate nuclear export of BAT3 proteins, cells were transfected with DNA constructs for 24 h, or cells remained untransfected. Subsequently cells were incubated with 10 ng leptomycin B (Axxora, Lörrach, Germany) per ml of culture medium for 2 h at 37°C. Cells were then fixed with methanol and stained for immunofluorescence microscopy.

## Results

### Alternative RNA splicing results in expression of several variant *BAT3* transcripts

Database evaluation of cDNA clones and expressed sequence tags (EST) suggested that several splicing events could occur in the coding region of the human *BAT3* transcript (Fig. 1A) [27]. Our aim was to identify naturally occurring BAT3 species. By sequencing *BAT3* cDNAs we found a not yet described coding sequence between exons 11 and 12, which was designated as exon 11B (Fig. 1A, Fig. S1). This exon sequence was found in PALSdb (putative alternative splicing) but strikingly not in the NCBI RefSeq databases [28]. According to this finding we suggest that in contrast to previous reports the *BAT3* gene is composed of 25 coding and one non-coding exon (exon 1). Evaluation of the *BAT3* gene sequence revealed that 11 of the 25 protein coding exons (grey), including exon 11B (black), could be deleted from the primary transcript while maintaining the reading frame (Fig. 1A). In cDNA databases it was also described that exon 1 and 7 can be alternatively spliced resulting in a different length of 271 bp or 247 bp for exon 1 and of 236 bp, 254 bp or 278 bp for exon 7.

By reverse transcription of melanoma cell line MelJuSo mRNA with a BAT3-specific primer we generated a cDNA pool as a source to screen for naturally occurring BAT3 splice variants. Since differential splicing of more than one exon could take place within one transcript, we amplified *BAT3* cDNAs using a forward primer starting in exon 2 adjacent to the ATG and a reverse primer at the end of exon 25 (compare MATERIALS AND METHODS). The obtained *BAT3* cDNAs were cloned into pcDNA 3.1-V5-His-TOPO vector containing a C-terminal V5 tag. We selected 100 clones with a *BAT3* insert of approximately 3.7 kb. To screen for the presence of the potentially spliced *BAT3* exons 5, 6, 9, 11, 11B, 17, 20, 21, 22, 23 and 24 (compare Fig. 1A), we generated ^32^P-labelled DNA probes specific for the respective exons. Detection of exons was conducted by colony hybridisation. To confirm the presence or absence of *BAT3* exons in the isolated cDNAs, the colony hybridisation assay was complemented by PCR with exon-flanking primer combinations (Fig. 1B and C, compare MATERIALS and METHODS). The screening result of 100 BAT3 cDNA clones is summarised in Table 1. Out of the eleven examined *BAT3* exon sequences we found exclusion of exons 5, 11B and 24. Deletion of exon 24 was recently described in cDNA databases (NCBI). The sequences of exons 6, 9, 11, 17, 20, 21, 22 and 23 were present in all of the 100 examined BAT3 cDNA clones. As a consequence of multiple splicing events, five BAT3 variants were detected in addition to BAT3 containing all exons (full-length BAT3), yielding altogether 6 variant BAT3 species. Deletion of exon 5 is a rare event and was found in only one of the inspected cDNA clones from MelJuSo cells. Single exon sequences 11B or 24 were absent in 23 or 22 of 100 cDNA clones. Two splicing events were observed in Δ5,11B (2 clones) and in Δ11B,24 (19 clones) variants. Thirty three clones contained all coding exon sequences (full-length). The product of frequencies of exon 11B (totally 44%) and of exon 24 (totally 41%) deletion yields a probability for the abundance of the double splicing event of about 18% (0.44×0.41), which is consistent with the number of 19/100 isolated Δ11B,24 BAT3 cDNA clones. One interpretation of this result is that there is no cooperative effect between splicing of exons 11B and 24 in the *BAT3* transcript.

**Table 1:** Frequency of spliced exons in MelJuSo cells.

spliced exons	frequency of alternative splicing*
exon 5	1/100
exon 11B	23/100
exon 24	22/100
exons 5 and 11B	2/100
exons 11b and 24	19/100
no alternative splicing	33/100

* A total number of 100 clones was analyzed.

**Figure 2:**
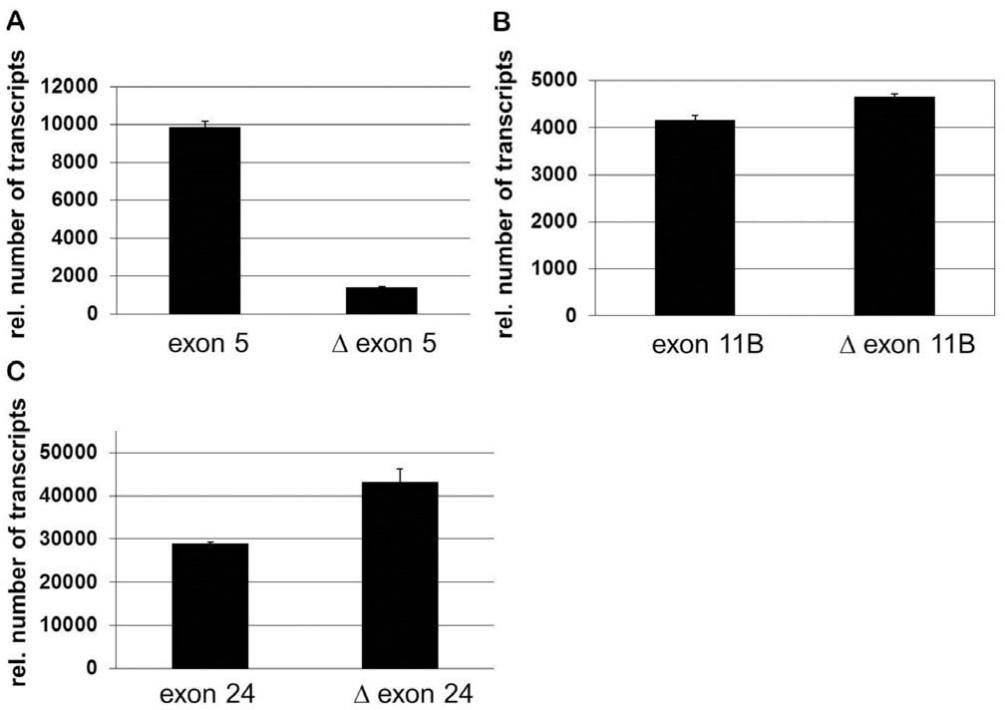
Real-time PCR analysis of BAT3 exons 5, 11B and 24. MelJuSo cells were subjected to RNA isolation and cDNA synthesis. BAT3 sequences were amplified by real-time PCR with specific oligonucleotides and examined for the presence of an exon (primers amplify DNA within the exon sequence) and for the absence of an exon (primers bind to exon boundaries). The presence or absence of exon 5 (**A**), exon 11B (**B**) and exon 24 (**C**) is shown as relative numbers of transcripts. Data are means ± SD of 5 experiments.

This study with a sample of 100 BAT3 cDNA clones does not exhaustively uncover the splice diversity of the *BAT3* gene but provides insight into naturally occurring BAT3 variants. To assess the splicing of BAT3 exons 5, 11B and 24 by a second method, we employed real-time PCR analysis. RNA was isolated from MelJuSo cells and reversely transcribed to the corresponding *BAT3* cDNA. *BAT3* exons 5, 11B and 24 were analyzed by monitoring splicing of sequences with primers hybridising respective exon boundaries (deleted) or within the exon sequence (undeleted). The result in Fig. 2A indicates that the exon 5 sequence is abundantly present in PCR products, whereas exon 11B is spliced in about half of the RNA (Fig. 2B). Deletion of the exon 24 sequence appears to be more frequent than the presence of exon 24 (Fig. 2C). The results in Fig. 2 are comparable to our data shown in Table 1. Both, colony hybridisation and real-time PCR confirm the presence of *BAT3* variants in MelJuSo cells. Some quantitative differences for the presence or absence of BAT3 exon sequences appear, in particular for exon 24. Reverse transcription of isolated mRNA yields a pool of cDNA species with different lengths, which impacts on DNA amplification by RT-PCR. Therefore, C-terminal sequences (Δexon 24, exon 24) are more efficiently transcribed to cDNA. In contrast, the colony hybridization assay was performed with 100 size-selected BAT3 cDNAs. An advantage of the colony hybridization screening is that complete BAT3 species with variable spliced exon sequences were identified.

**Figure 3:**
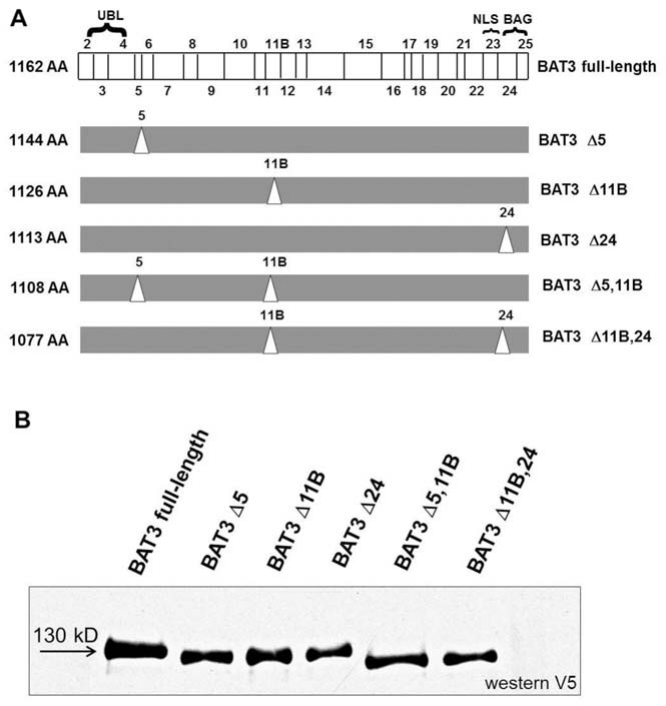
Identification of BAT3 splice variants. **A.** Schematic presentation of BAT3 variants. BAT3 full-length containing exons 2 to 25 is shown on top (translation starts with exon 2). Identified BAT3 variants with deleted sequences are shown below. Deleted exons are indicated by triangles. Designation of the variants is shown on the right and the calculated number of amino acids (AA) of the BAT3 proteins is indicated on the left. UBL: ubiquitin-like domain, NLS: nuclear localization signal, BAG: Bcl-2 associated athanogene-domain, **B.** Expression of BAT3 isoforms. COS-7 cells were transfected with V5-tagged BAT3 cDNAs, lysed, separated by SDS-PAGE and immunoblotted for BAT3 with anti V5 antibody. BAT3 variants are indicated on the top and a molecular weight marker is shown on the left. Compared to the full length BAT3 with a molecular weight of about 130 kDa, the calculated sizes of the variants are the following: BAT3 Δ5 (128 kDa), BAT3 Δ11b (127 kDa), BAT3 Δ24 (125 kDa), BAT3 Δ5, 11b (125 kDa) and BAT3 Δ11b, 24 (122 kDa).

Taken together, we isolated six BAT3 variants from the 100 inspected cDNA clones (Fig. 3A). Two presumably rare BAT3 species, Δ5,11B,24 and Δ5,24, which were additionally expected by differential splicing events, were not detected. All variants described here contain the short form of exon 7 with 236 bp (NCBI). To demonstrate expression of the six BAT3 isoforms, COS-7 cells were transfected with the indicated BAT3 cDNA constructs. The viability of cells with over-expressed BAT3 isoforms is not affected, as could be suspected from a role of BAT3 in apoptosis (not shown). Cell extracts were separated by SDS PAGE and immunoblotted for the V5-tagged BAT3 isoforms. Fig. 3B displays the variant BAT3 products, which migrate at approximately 120 to 130 kDa. To investigate whether endogenous isoforms of the BAT3 protein exist, we performed a SDS gradient gel electrophoresis of a MelJuSo cell lysate (Fig. S2). The lysate was immunoblotted for BAT3 using a polyclonal anti-BAT3 serum. We detected two protein bands demonstrating diversity of naturally occurring BAT3 protein.

### Distinct subcellular localization of BAT3 in various cell types

**Figure 4:**
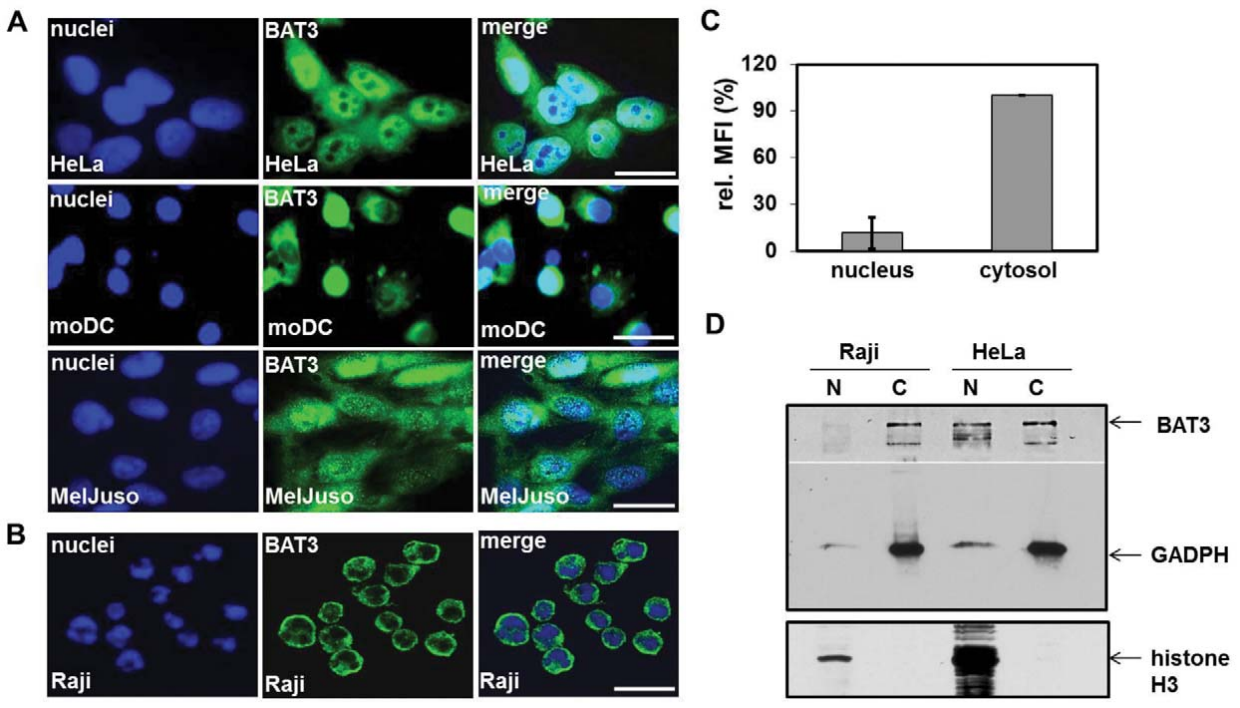
Subcellular localization of endogenous BAT3 in four cell types. HeLa cells (Adenocarcinoma), the human melanoma cell line MelJuSo, the B lymphoma Raji and monocyte-derived dendritic cells (moDCs) were plated on coverslips and stained for BAT3 using a polyclonal serum against a C-terminal peptide (middle lane). Cell nuclei (left lane) were visualized with DAPI (A) or 7AAD (B). Merged images are shown in the right lane. **A.** Immunofluorescence staining was evaluated with a standard fluorescence microscope and **B.** by confocal microscopy. Scale bars = 10 µm. **C.** Nuclear and cytosolic staining of endogenous BAT3 in Raji cells was evaluated in 10 single cells using ImageJ. MFI, mean fluorescence intensity per region of interest **D.** Western blot analysis of subcellular fractions from Raji and HeLa cells. Nuclei (N) and cytoplasm (C) were separated by SDS-PAGE and immunoblotted for BAT3, GADPH (cytosolic marker) and histone H3 (nuclear marker).

In previous reports the BAT3 protein was found in the nucleus and in the cytosol [9], [10], [11]. We inspected expression of endogenous BAT3 in various cell lines and in primary cells by immunofluorescence microscopy. For detection of BAT3, cells were plated on coverslips and stained with antiserum against BAT3. Expression of BAT3 was visualized with AlexaFluor488-conjugated antibody and examined by fluorescence microscopy. We monitored four human cell types, HeLa (Adenocarcinoma), monocyte-derived dendritic cells (moDC), MelJuSo cells (Melanoma) and the B lymphoma cell line Raji for intracellular expression of BAT3. The left panel of Fig. 4 A and B shows nuclear and the middle panel BAT3 staining. On the right, both images were merged. HeLa, moDCs and MelJuSo cells display both cytosolic and nuclear staining of BAT3, albeit nuclear staining of moDCs and MelJuSo cells is reduced compared to HeLa cells (Fig. 4A). Due to the small size of Raji cells evaluation of staining with standard fluorescence microscopy proved difficult. To monitor subcellular labelling of Raji cells, we applied confocal microscopy for detection of BAT3 (Fig. 4B). When opposed to the other examined cell types, Raji cells mainly show cytosolic staining of BAT3. Nuclear and cytosolic expression of BAT3 in Raji cells was quantified by calculating the mean fluorescence intensity (MFI) in the nucleus and in the cytosol (Fig. 4C). Compared to nuclear BAT3 staining we detected a 10-fold increase of signal intensity in the cytosol. To confirm the predominant cytosolic localization of BAT3 in Raji cells, we prepared subcellular fractions of nuclei and cytoplasm from Raji and HeLa cells. Western blotting of nuclear and cytosolic fractions showed that the BAT3 protein was only detected in the cytosol of Raji cells, but in nuclear and cytosolic fractions of HeLa cells (Fig. 4D). In addition, the blot was stained with cytosolic (GADPH) and nuclear (H3) markers. For a quantitative evaluation of the four examined cell types, we counted subcellular staining of 100 cells (Table 2). HeLa cells showed both, nuclear and cytosolic staining for BAT3. MoDCs and MelJuSo displayed exclusively cytosolic localization of BAT3 in 74 and 54% and both, nuclear and cytosolic staining in 26 and 46% of the inspected cells. Counting of Raji cells revealed almost no nuclear staining of BAT3, but entire cytosolic localization. Our studies indicate a cell type specific distribution of BAT3, either in the cytosol or in both, the nucleus and the cytosol. Since the *BAT3* gene gives rise to expression of several RNA splice variants, the assessed intracellular distribution of BAT3 could emerge from a variable expression of BAT3 isoforms.

**Table 2:** Subcellular localization of endogenous BAT3 in % of cells.

cell type	unique cytosolic staining	nuclear and cytosolic staining
HeLa	0	100
moDC	74	26
MelJuSo	54	46
Raji	100	0

### Cell type-specific expression pattern of *BAT3* splice variants

To explore whether expression of *BAT3* variants is tissue-specific, we inspected the splicing pattern in Raji, HeLa and in primary moDCs. From each cell type, 20 *BAT3* cDNA clones with a size of 3.7 kb were isolated. Subsequently, the exon composition of these cDNA clones was inspected by PCR (compare Fig. 1B and C). The result from evaluation of BAT3 cDNA clones is shown in Table 3. In 20 cDNA clones derived from Raji cells, we found two and from HeLa and moDCs, 9 and 11 clones, which show no deletion of exon sequences and therefore encode full-length *BAT3*. Exclusion of the exon 11B sequence was detected in 17 of the Raji, but only in 3 of the moDC cDNA clones. In eight of the HeLa cDNA clones the sequence of exon 11B is omitted. Splicing of exon 24 occurred in 7, 6 or 7 clones derived from the B lymphoma, from HeLa cells or from moDC derived BAT3 cDNAs. Alternative splicing of exon 5 was not found in any of the 60 (3×20) inspected cDNA clones. Sequences derived from exons 5, 6, 9, 11, 17, 20, 21, 22 and 23 (compare Fig. 1A) were detected in all examined cDNA clones (not shown). Our screening for tissue-specific expression of *BAT3* splice variants yielded a significant difference in the number of variants, particulary in Raji cells and moDCs. The B lymphoma Raji contains *BAT3* variants mainly without exon 11B, whereas full-length and deletion of exon 24 were predominantly found in moDCs as a source of primary cells.

**Table 3:** Tissue specific expression of *BAT3* splice variants.

*BAT3* variant	Raji cells	HeLa cells	moDCs
full-length	2	9	11
Δ 11B	11	5	2
Δ 24	1	3	6
Δ 11B,24	6	3	1

The presence of BAT3 exon 11B splice variant in different cell types was further investigated by real-time PCR analysis (ΔΔC_t_ method with actin as endogenous control). Therefore, mRNAs of MelJuSo cells, primary monocytes and of T lymphoid CEMC7 cells were tested for exon 11B sequences using exon 11B-specific oligonucleotides (Fig. S3). The relative levels of exon 11B transcripts in monocytes and CEMC7 cells were calculated in relation to those in MelJuSo cells (RQ = 1). In CEMC7 we found reduced amounts of exon 11B sequences compared to MelJuSo cells.

### Subcellular localization of BAT3 splice variants in transfected HeLa cells

**Figure 5:**
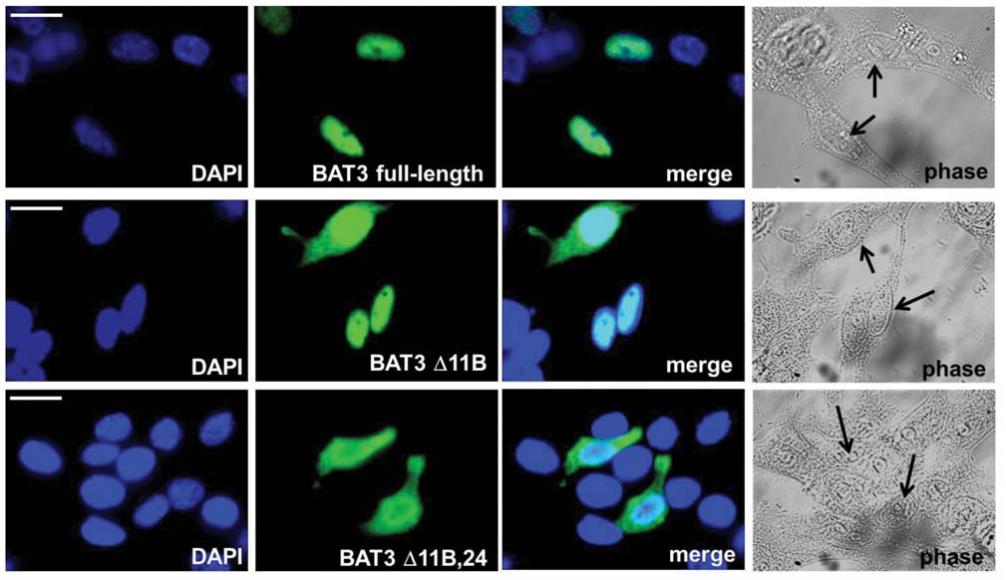
Subcellular localization of BAT3 variants in transfected HeLa cells. Cells were transfected with BAT3 splice variants and stained after 24 hours with a monoclonal V5 antibody for evaluation by immunofluorescence microscopy. Left panel displays DAPI staining, second panel BAT3 staining, third panel merging of images and right panel shows corresponding phase contrast images. The displayed transfected cells show examples for nuclear (BAT3 full-length, upper panel), for nuclear and cytosolic (BAT3 Δ11B, middle panel) and for enhanced cytosolic staining (BAT3 Δ11B, 24, lower panel). Scale bars = 10 µm.

To inspect the intracellular localization of BAT3 variants, we transfected HeLa cells with each of the six V5-tagged *BAT3* cDNA species. Cells were plated on coverslips and stained with a monoclonal antibody against the V5 tag. Expression of BAT3 was visualized with AlexaFluor488-conjugated antibody and inspected by fluorescence microscopy. Fig. 5 displays three examples of transfected splice variants with a characteristic subcellular distribution. The upper panel shows that full-length BAT3 is almost exclusively expressed in the nucleus of HeLa cells. BAT3 Δ11B (middle panel) shows nuclear expression but in addition nuclear and cytosolic staining. HeLa cells transfected with the Δ11B,24 variant (lower panel) display enhanced cytosolic labelling of BAT3 in comparison to cells transfected with BAT3 Δ11B. Table 4 displays an evaluation of the subcellular distribution of 6 variant BAT3 proteins in 100 cells. In HeLa cells, BAT3 full-length and BAT3 Δ24 were mainly found in the nucleus. When single exons 5 or 11B were deleted, we detected in about 50% of the cells nuclear localization, 10% with sole cytosolic, and in almost 40% of the cells staining of both, the nucleus and the cytoplasm. The BAT3 variants Δ5,11B and Δ11B,24 showed sole cytosolic expression for 14 or 25% of the transfected cells and for 46 or 63% staining of both, nucleus and cytosol. Comparable results were obtained by expression of BAT3 variants in MelJuSo cells (data not shown), suggesting that expression of splice variants yields the observed subcellular localization of BAT3 in different cell types.

**Table 4:** Subcellular localization of the BAT3 variants in % of transfected HeLa cells.

BAT3 variant	unique nuclear staining	unique cytosolic staining	nuclear/cytosolic staining
full-length	85	0	15
Δ 5	51	11	38
Δ 11	55	9	36
Δ 24	90	0	10
Δ 5,11B	40	14	46
Δ 11B,24	12	25	63

### Export of BAT3 from the nucleus to the cytosol is impaired by leptomycin B treatment

**Figure 6:**
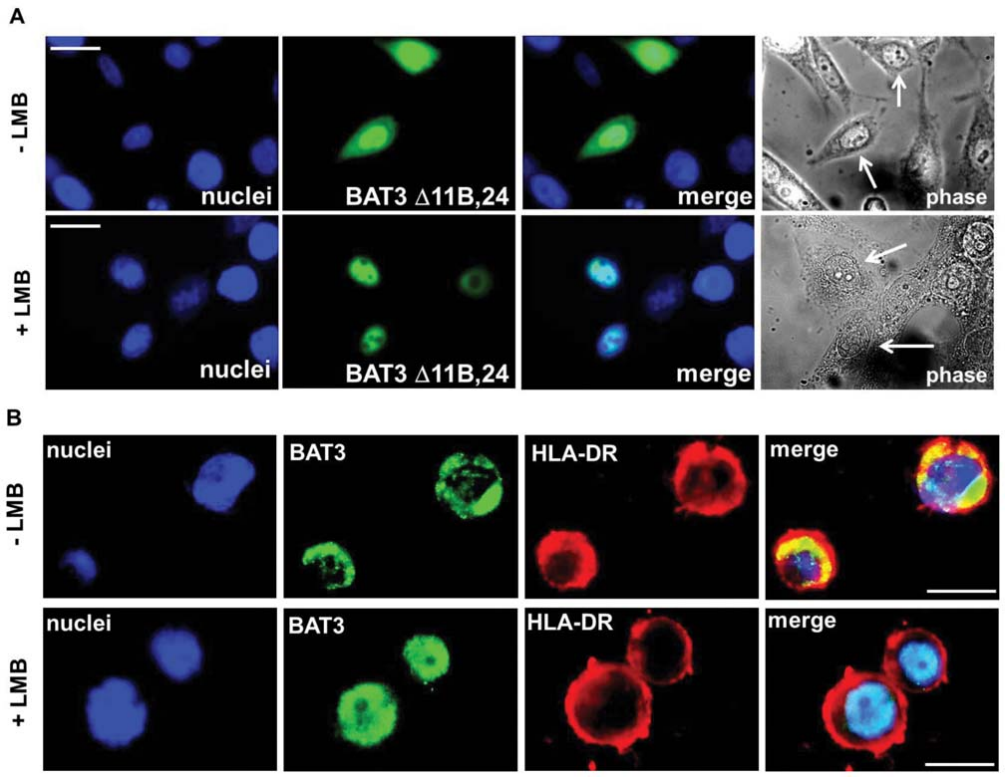
Impact of leptomycin B treatment on the subcellular localization of transfected BAT3 splice variants and of endogenous BAT3. **A.** HeLa cells transfected with BAT3 Δ11B,24 were cultured on coverslips in the presence (lower panel) or absence (upper panel) of leptomycin B (LMB) for 2 h. Cells were subsequently stained with V5 mAb and inspected with standard immunofluorescence microscopy (second panel). Left panel shows DAPI stained nuclei, third panel merging of images and right panel displays phase contrast images. Scale bars = 10 µm. **B.** Raji cells were cultured for 2 h in the presence (lower panel) or absence (upper panel) of LMB and then plated on coverslips. Cells were subsequently stained with the polyclonal anti-BAT3 serum and with ISCR3 mAb (HLA-DR) for evaluation by immunofluorescence microscopy. Left panel shows DAPI staining, second panel staining for BAT3, third panel staining for HLA-DR and images were merged in the right panel. Scale bars = 5 µm.

Since the identified BAT3 splice variants contain a nuclear localization signal at the C-terminus encoded in exon 23 [18], we assume that all variants should be translocated from the cytosol to the nucleus by a similar mechanism. To examine whether the observed elevated cytosolic expression of BAT3 splice variants without exon 11B may depend on nuclear export, we employed the inhibitor leptomycin B (LMB), which impairs the Crm1-dependent nuclear export of proteins [29]. HeLa cells were transfected with V5-tagged BAT3 cDNA species and incubated for 2 h with LMB. The subcellular distribution of transfected BAT3 variants was inspected by immunofluorescence microscopy. An example is shown for BAT3 Δ11B,24 (Fig. 6A). BAT3 Δ11B,24 displays nuclear and cytosolic labelling, but upon incubation with LMB for 2 h sole nuclear staining was obtained. The other BAT3 variants, which show cytosolic expression, also localize to the nucleus after treatment with LMB (data not shown).

As a consequence of impaired nuclear export of BAT3 upon LMB treatment, we expected that the B lymphoma cell line Raji, which displays prominent cytosolic expression of BAT3, should strongly respond to incubation with LMB. Raji cells were plated on coverslips, treated for 2 h with LMB and stained for BAT3 (Fig. 6B). To visualize the extranuclear space of Raji cells, we performed a co-staining of BAT3 and HLA-DR (Fig. 6B). As a result of LMB incubation, we detected predominant nuclear staining of endogenous BAT3 in Raji cells (Fig. 6B, lower panel), compared to non-treated cells (upper panel). Thus, when nuclear export is blocked in Raji cells, nuclear import of BAT3 yields complete nuclear localization.

LMB inhibition indicates that the nuclear export of BAT3 depends on the Crm1 pathway. Crm1 mediated nuclear export of BAT3 is impaired by a sequence encoded by exon 11B, because BAT3 variants deleted of exon 11B are frequently located in the cytoplasm, whereas BAT3 full-length and Δ24 are predominantly expressed in the nucleus. Therefore, the dual localization of BAT3 isoforms in the cytosol and the nucleus may be controlled on the level of nuclear export.

## Discussion

Differential splicing of transcripts occurs in about 80% of genes in the human genome [30]. Alternative RNA splicing results in various protein species from the same primary transcript but may maintain structural elements (e.g. ligand-binding domains) in the variant splice products. A set of such splice variants can simultaneously exert unique and diverse functions. Regulation of splicing pattern is based on phosphorylation, shuttling of splicing factors between the nucleus and the cytosol, assembly of the splicing complex, the influence of extracellular stimuli on specific RNA-binding proteins and other stages of pre-mRNA processing [30], [31], [32]. Our data suggest that alternative pre-mRNA splicing also contributes to the extensive diversity of a gene in the HLA complex, which encodes the class III protein BAT3. BAT3 is capable of multiple functions and differential splicing of *BAT3* pre-mRNA suggests that the splicing pattern implicates various cellular activities. Since not all splice variants are translated to proteins, expression of BAT3 isoforms should be adjusted to cellular functions.

In our study, we discovered six BAT3 splice variants which display a cell type specific expression pattern in human cell lines and in primary cells. Moreover, we detected the novel alternatively spliced *BAT3* exon sequence 11B. In order to investigate whether the splicing pattern of BAT3 correlates with cytosolic or nuclear/cytosolic staining of the endogenous protein in examined cell types, we found that some BAT3 isoforms are preferentially localized in the nucleus (BAT3 full-length and BAT3 Δ24), whereas BAT3 deleted of exon 11B is frequently located in the cytoplasm (Fig. 5). The examined cells express similar levels of BAT3 (not shown). Therefore, the subcellular distribution of BAT3 reflects properties of the individual isoforms or of associated cofactors. Previous studies suggested that BAT3 is dominantly targeted to the nucleus [11], [18]. In recent years, BAT3 was shown to have in addition multiple roles in the cytosol [13], [15]. Shuttling between the nucleus and the cytoplasm is mediated by import and export signals [33]. The import/export cycle determines the subcellular localization of proteins. In most cases, the nuclear import is a rapid process. Factors binding to a nuclear localization signal (NLS) promote translocation of the cytosolic protein to the nucleus. The BAG domain encoded by exons 24 and 25 of the *BAT3* gene is in close proximity to the NLS encoded by exon 23 [7], [18]. It is conceivable that masking of exons such as 24 and 25 by a ligand impacts on nuclear localization of the BAT3 polypeptide. The BAG domain was shown to interact with the ATPase domain of the heat shock protein Hsc70/HSP70 family [34], [35]. Scythe/BAT3 directly inhibits Hsp70 protein folding activity [35]. Interaction with HSP70 possibly affects the recruitment of NLS binding factors and thereby regulates nuclear import of BAT3. Our results indicate that the presence or absence of exon 24 has no impact on the nuclear localization of the BAT3 protein since both, full-length protein and BAT3 Δ24 are predominantly expressed in the nucleus. Shuttling of BAT3 between the nucleus and the cytoplasm may also require a nuclear export signal (NES), which was proposed to localize in exon 8 [11]. Our data suggest that nuclear export of BAT3 is mediated by the Crm1 pathway and may be influenced by the exon 11B sequence. BAT3 isoforms lacking exon 11B show intense cytosolic localization, which is abrogated by LMB treatment. A possible role of specific exon sequences for subcellular distribution of the BAT3 isoforms has to be further explored. It is conceivable that the presence or absence of sequences adjacent to the NES impacts on the molecular structure of BAT3 leading to a modified accessibility of the NES. We demonstrated that 2 h of LMB treatment completely abolishes cytosolic BAT3 expression, indicating that nuclear export rather than a delayed import determines the cytosolic localization of BAT3.

We suppose that cell type-specific expression of BAT3 arises from the cell type-specific pattern of splice variants with a distinct subcellular localization of BAT3 isoforms. Isolation of cDNA clones from HeLa cells indicated expression of four different BAT3 variants (Table 3). Subcellular distribution of the four BAT3 variants is consistent with cytosolic and nuclear staining of endogenous BAT3 in HeLa cells (summarised in Table 2). The B lymphoma cell line Raji mainly exhibits cytosolic staining of BAT3 as shown in Fig. 4B–D. Isolation of the numerous BAT3 variants with deleted exon 11B from Raji cells is in agreement with cytosolic staining of the isoforms in transfected HeLa cells. However, expression of two BAT3 variants, BAT3 full-length and BAT3 Δ24 (Table 3) should yield more nuclear staining of the B lymphoma cells than observed by immunofluorescence microscopy. This would also be predicted for moDCs, which show substantial cytosolic expression but high levels of BAT3 full-length and Δ24 (Table 2 and 3). One would expect a higher level of nuclear staining for BAT3 in moDCs, if the intracellular localization is solely dependent on expression of the BAT3 variants. Thus, tissue-specific factors may additionally impact on the intracellular localization of BAT3.

Shuttling of proteins between the nucleus and the cytoplasm was described as a mechanism for regulation of apoptosis and to maintain a basal activity of transcription factors by signalling molecules [36], [37], [38]. In concert with this mechanism, BAT3 was suggested to act as a nucleus-cytoplasm shuttling protein regulating apoptotic cell death induced by papillomavirus binding factor (PBF) in human osteosarcoma [39]. It is conceivable that BAT3 variants which localize in the nucleus or in the nucleus/cytosol have distinct roles in controlling folding and activity of various signalling molecules regulating apoptosis, cell cycle and proliferation. Physical interaction with BAT3 has been demonstrated for apoptosis modulating protein X1-1, for AIF and for reaper [11], [40], for cell division controlling human small glutamine-rich TPR-containing protein (hSGT) and for a MAK-related kinase [34], [41]. Previously, BAT3 was identified as a substrate of the apoptosis-inducing caspase-3. The cleavage site for caspase-3 was found at the C-terminus of BAT3 encoded by exon 22 [42]. Three novel proteins, which contain domains that are involved in protein degradation, were detected by yeast two-hybrid screening to interact with the N-terminus of BAT3 [43]. Recently, it was demonstrated that BAT3 forms a complex with TRC35 and Ubl4A, which is recruited to ribosomes and acts as a chaperone that channels tail-anchored proteins to the TRC40 insertion pathway [13]. In addition, this BAT3 complex was shown to link targeting and degradation of mislocalized proteins (MLPs) by recognizing hydrophobic domains that distinguish MLPs from potential cytosolic proteins [15]. Moreover, BAT3 was found to be involved in endoplasmic reticulum-associated degradation of misfolded proteins by maintaining polypeptide solubility to avoid aggregation before reaching the proteasome [14]. In this process, one role of TRC35 is to retain BAT3 in the cytosol. At present, it is unclear whether retention of the BAT3 isoforms correlates with interaction to TRC35. Recently, we demonstrated that BAT3 chaperones the HLA class II transactivator (CIITA) and thereby modulates the expression of components of the HLA class II antigen presentation pathway [25]. Another study with immunologic implications revealed that BAT3 serves as a target during the course of *Legionella pneumophila* infection and is modulated by multiple translocated bacterial substrates [44], possibly to escape antigen presentation by HLA class II.

In conclusion, variants of BAT3 may differ in their network of interactions with other proteins. Proposing that BAT3 stabilizes and targets cellular proteins, it will be important to identify substrates and interaction profiles of the various BAT3 proteins.

Aberrant alternative pre-mRNA splicing plays a role in human pathologies. Experimental and computational studies revealed examples for specific splice variants that are detectable only in cancerous tissues [45], [46]. Diseases such as Myasthenia gravis, thymus hyperplasia and some adverse drug reactions were linked to the *BAT3* locus [47], [48]. A rare *BAT3* allele was found in three “diabetogenic” HLA haplotypes [49]. It is not yet uncovered, whether abnormal splicing in the *BAT3* locus is linked to autoimmunity. Evaluation of the diversity of BAT3 transcripts may help to understand the role of this protein in various cellular functions as well as under pathological conditions.

## Supporting Information

Figure S1 **Exon-intron-organization of the** ***BAT3*** **gene between exons 10 and 13.** *BAT3* exons 11, 11B and 12 are highlighted in bold.
(TIF)

Figure S2 **SDS gradient gel electrophoresis of a MelJuSo lysate.** A MelJuSo lysate was separated by SDS gradient electrophoresis (7–12%) and transferred to nitrocellulose membrane, which was probed with rabbit anti-BAT3 serum.
(TIF)

Figure S3 **Relative quantification of exon 11B expression.** MelJuSo cells, primary human monocytes and the lymphoid cell line CEMC7 were subjected to mRNA isolation and cDNA synthesis. The presence of exon 11B transcripts was analyzed by real-time PCR (ΔΔC_t_ method) using indicated oligonucleotides (Materials and Methods). Relative quantification (RQ) of exon 11B transcripts is shown for three independent experiments (1–3) and was calculated on the exon 11B level in MelJuSo cells.
(TIF)
