# Supplementary figures and images for: A Novel BAT3 Sequence Generated by Alternative RNA Splicing of Exon 11B Displays Cell Type-Specific Expression and Impacts on Subcellular Localization
Source: PLoS One. 2012 Apr 25;7(4):e35972. doi: 10.1371/journal.pone.0035972 (PMC3338477; doi:10.1371/journal.pone.0035972)

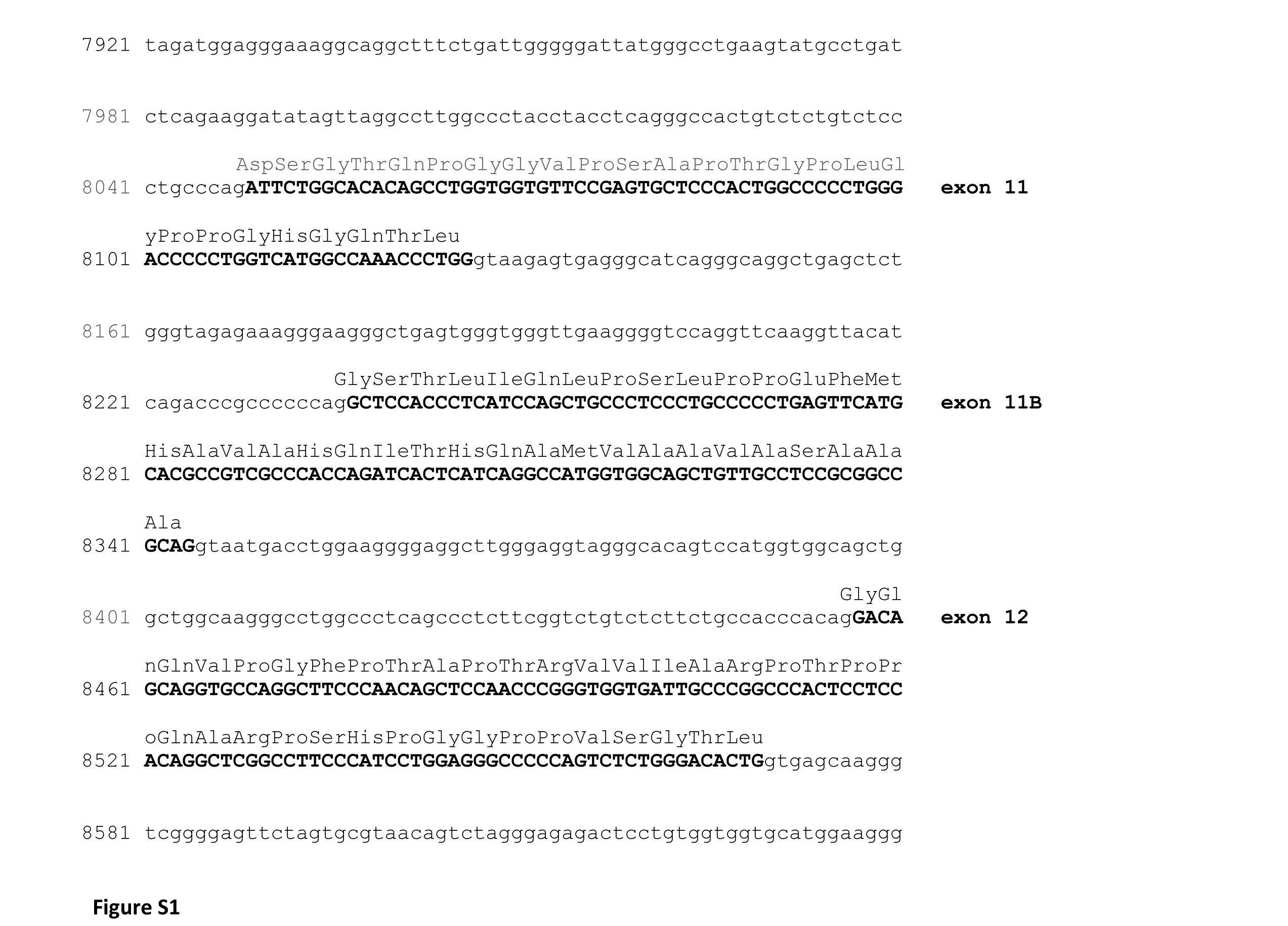

Supplement: Figure S1 — Exon-intron-organization of the BAT3 gene between exons 10 and 13. BAT3 exons 11, 11B and 12 are highlighted in bold. (TIF) [file pone.0035972.s001.tif]

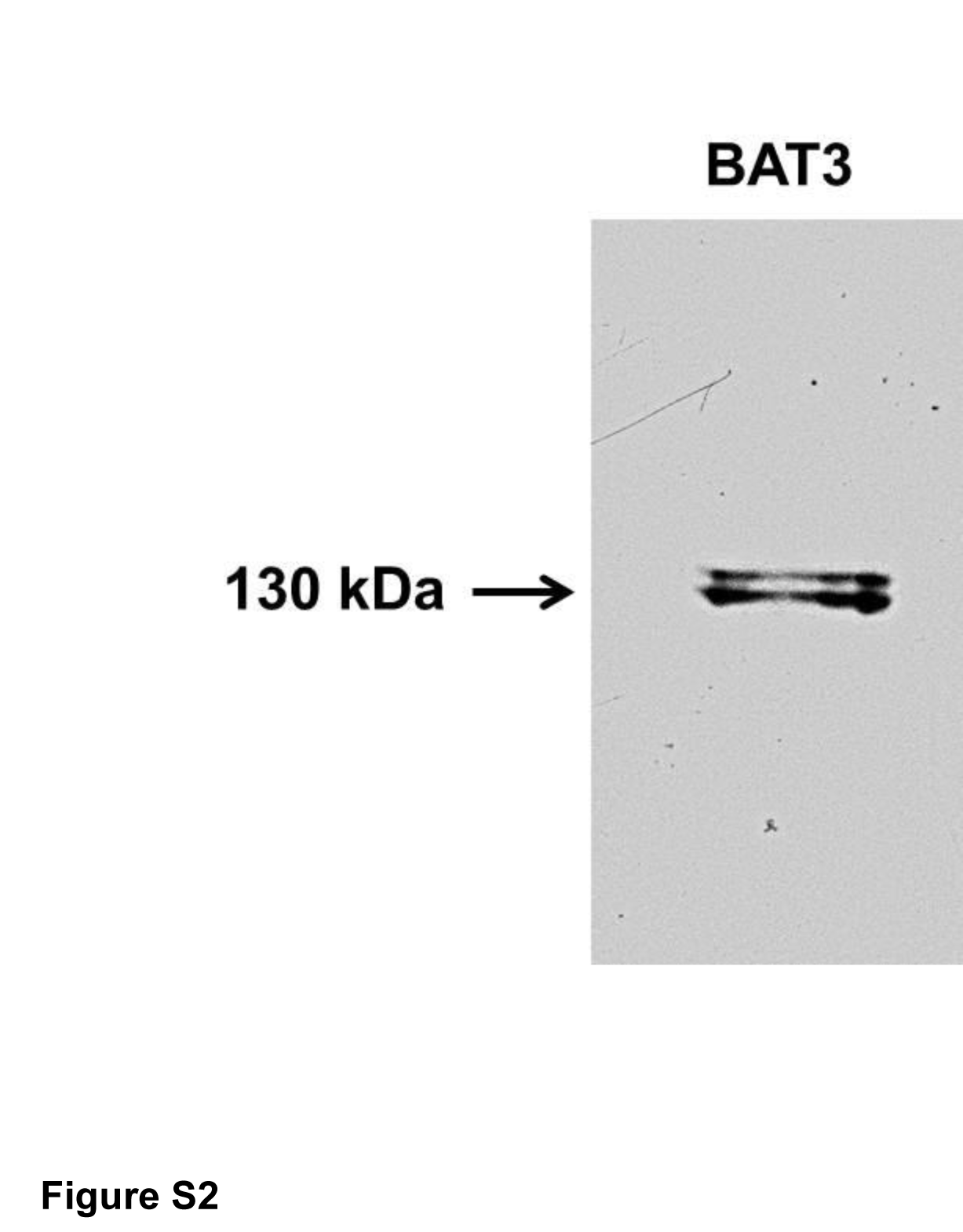

Supplement: Figure S2 — SDS gradient gel electrophoresis of a MelJuSo lysate. A MelJuSo lysate was separated by SDS gradient electrophoresis (7–12%) and transferred to nitrocellulose membrane, which was probed with rabbit anti-BAT3 serum. (TIF) [file pone.0035972.s002.tif]

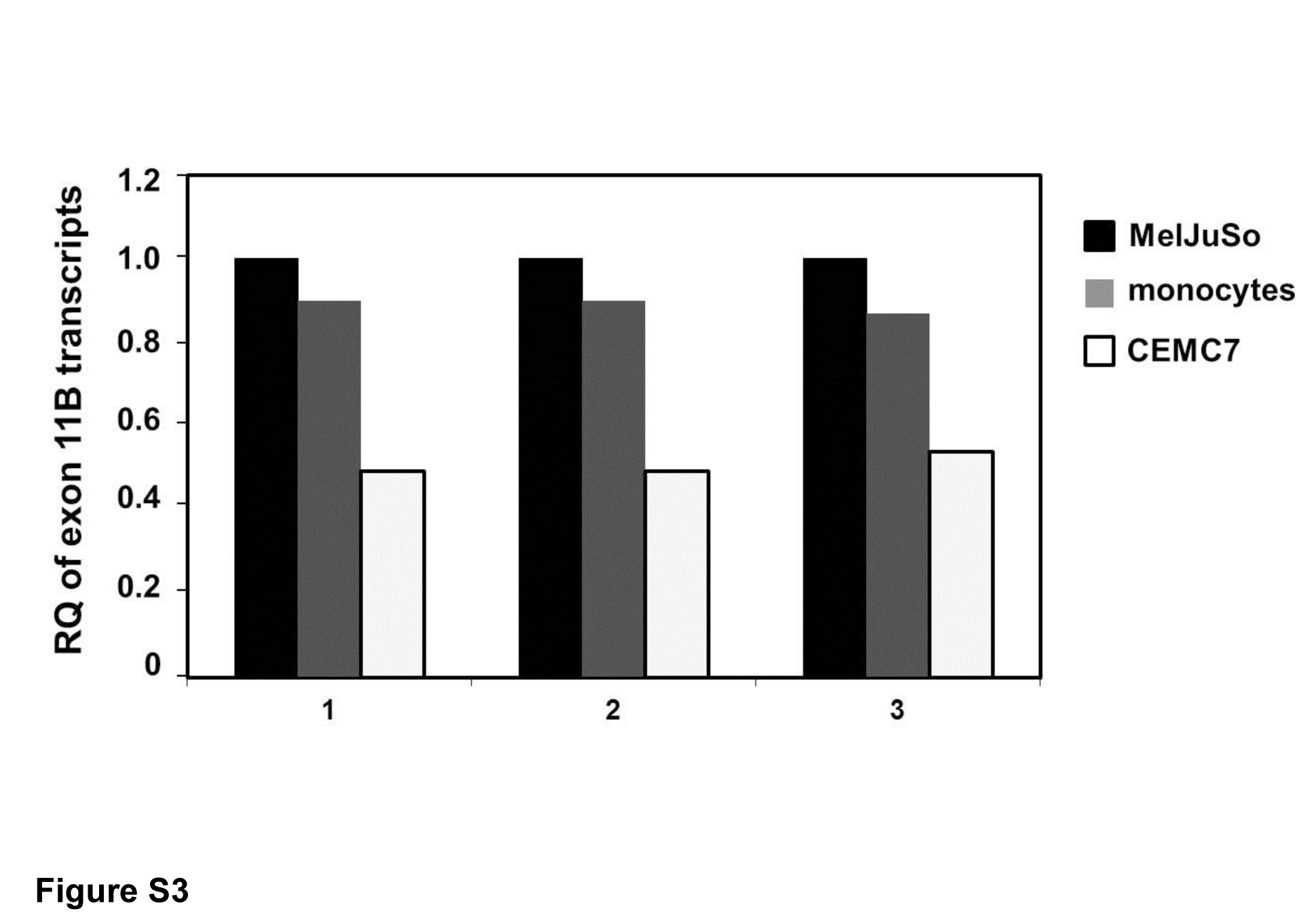

Supplement: Figure S3 — Relative quantification of exon 11B expression. MelJuSo cells, primary human monocytes and the lymphoid cell line CEMC7 were subjected to mRNA isolation and cDNA synthesis. The presence of exon 11B transcripts was analyzed by real-time PCR (ΔΔCt method) using indicated oligonucleotides (Materials and Methods). Relative quantification (RQ) of exon 11B transcripts is shown for three independent experiments (1–3) and was calculated on the exon 11B level in MelJuSo cells. (TIF) [file pone.0035972.s003.tif]
